# Effects of ethanol leaf extract of *Spondias mombin* on scopolamine-induced hippocampal neurodegeneration in adult male Wistar rats: evidence from behavioral, biochemical, histological, and immunohistochemical analyses

**DOI:** 10.3389/fncel.2026.1855376

**Published:** 2026-06-16

**Authors:** Temidayo Matthew Ale, Babatunde Elijah Arayombo, Alfred Wehdoe Sloh

**Affiliations:** Department of Anatomy and Cell Biology, Obafemi Awolowo University, Ile-Ife, Osun, Nigeria

**Keywords:** hippocampus, neurodegeneration, *Spondias mombin*, stratum alveus, Wistar rat

## Abstract

Previous studies have demonstrated that phytochemicals present in *Spondias mombin* leaves possess neuro-regenerative and neuroprotective potentials. This study therefore evaluated the effects of ethanol leaf extract of *Spondias mombin* (ELESM) on the hippocampus of adult male Wistar rats with scopolamine-induced neurodegeneration using biochemical, behavioral, histological, and immunohistochemical assessments. This study was conducted for a period of 71 days. Forty-two adults male Wistar rats (200 ± 20 g) were randomly assigned into six groups (*n* = 7). Group I received normal saline (1 mL/kg) orally for 10 weeks. Neurodegeneration was induced in Groups II–VI by intraperitoneal administration of scopolamine (1 mg/kg) for two weeks. Twenty-four hours after induction, Group II received normal saline (1 mL/kg) orally, Group III received donepezil (5 mg/kg) orally, while Groups IV–VI received ELESM (200, 400, and 600 mg/kg) respectively for eight weeks. Result showed that scopolamine administration significantly increased acetylcholinesterase activity, reduced antioxidant status, and promoted inflammation. Behavioral assessments showed reduced locomotor activity, anxiety-like behavior accompanied with impaired working memory. Histological findings demonstrated features of neurodegeneration, including reduced neuronal density in the dentate gyrus and a decreased proportion of healthy neurons in the cornu ammonis subfields. White matter integrity in the alveus was compromised, appearing distorted and de-compacted. Immunohistochemical analysis further revealed astrocytic reactivity in response to oxidative stress. These findings indicate that scopolamine triggered molecular and structural alterations that persisted beyond the induction period. However, treatment with Donepezil and ELESM 200 mg/kg, 400 mg/kg and 600 mg/kg produced significant anticholinesterase, antioxidant, and anti-inflammatory effects, restoring these protein levels to near normal. Donepezil and ELESM improved cognitive and memory functions and attenuated anxiety-like behavior. Histological integrities of the hippocampus subfields were preserved, while immunohistochemistry showed modulation of astrocytic glial fibrillary acidic protein expression. Chronic scopolamine administration caused long term impairments, but ELESM demonstrated neuroprotective effects against scopolamine-induced neurodegeneration, likely mediated through its anticholinesterase, antioxidant, and anti-inflammatory properties in adult male Wistar rats.

## Introduction

Neurodegeneration is a pathological process involving oxidative stress, inflammation, and neuronal network dysfunction, culminating in neuronal loss, structural damage, and functional impairment. Neurodegenerative diseases are characterized by a distinct set of hallmarks, including pathological protein aggregation, dysfunction of synapses and neuronal networks, disrupted proteostasis, cytoskeletal abnormalities, altered energy metabolism, genetic and transcriptomic defects, neuroinflammation, and neuronal cell death (Wilson et al., 2023). These disorders represent a major global health challenge, affecting millions of individuals worldwide, with prevalence expected to increase significantly in developing countries, including Nigeria (Oladele et al., 2020).

The development and progression of neurodegenerative diseases are influenced by several factors, such as aging, genetics, environmental exposure, and trauma, although aging remains the most significant risk factor (Lamptey et al., 2022; Liu et al., 2022). Neurodegenerative disorders comprise a broad spectrum of conditions, including Alzheimer’s disease (AD), Parkinson’s disease (PD), frontotemporal dementia, primary tauopathies and many others (Wilson et al., 2023). Among these disorders, Alzheimer’s disease is the most prevalent and devastating form of dementia worldwide.

Alzheimer’s disease (AD) is the 6th leading cause of mortality worldwide, with more than a hundred thousand deaths recorded in 2019; moreover, more than 55 million people worldwide are living with dementia, with approximately 60% of the affected belonging to low- and middle-income nations (Naheed et al., 2023). AD is a pathology whose progression consists of neuronal degeneration, especially the cholinergic ones. There is strong genetic and clinical evidence supporting that the deposition of amyloid-beta (Aβ) which is the product of beta-amyloid precursor protein (APP) and neurofibrillary tangles (NFTs), which consist of abnormally phosphorylated microtubule-associated tau protein are the major pathological signs of the disease (Sharma et al., 2017). As reported by Boisvert et al. (2018), ‘the pathogenesis of AD is associated with histo-cellular, anatomical, and neurochemical changes in the brain, leading to reduced neuronal activity, cortical neuron loss, synaptic disconnection, and memory impairment’. These pathological changes affect mainly the prefrontal cortex and hippocampus which are important areas in processing memories.

Due to its critical role in learning, memory formation, and cognitive processing, the hippocampus (Figure 1b) is particularly vulnerable to the pathological changes associated with AD. A bilateral damage of the hippocampus may result in anterograde amnesia; a condition in which the brain fails to establish new long-term memory (Chauhan et al., 2021). A number of neurological conditions like AD and PD, are characterized by hippocampal damage which impairs memory and cognitive function. The loss of neuron and the alteration of the structures in subfields of the hippocampus is a solid biomarker to predict AD (Xu et al., 2023). The dysfunction of synapses in the hippocampus is one of the first pathological signs of the AD, even prior to the appearance of amyloid plaques and the main cause of memory loss (Shankar and Walsh, 2009).

**Figure 1:**
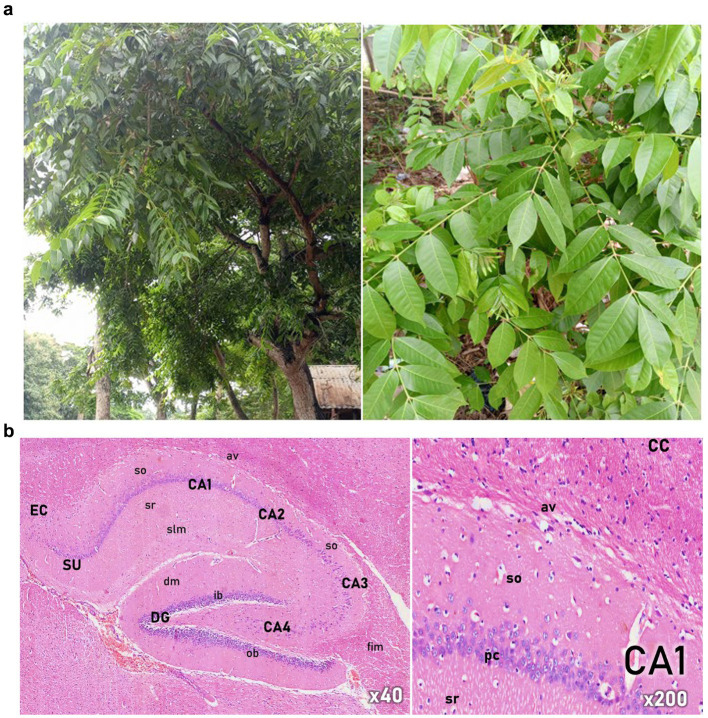
**(a)** The hog plum tree (*Spondias mombin* L.). **(b)** Histology of a rat’s hippocampus (x40) and its CA1 subfield (x200). CA, cornu ammonis; DG, dentate gyrus; EC, entorhinal cortex; SO, stratum oriens; SR, stratum radiatum; SLM, stratum lacunosum moleculare; FIM, fimibrae; IB, internal blade of dentate gyrus; DM, molecular layer of dendate gyrus; OB, outer blade of dentate gyrus; CC, corpus callosum; SU, subiculum; AV, stratum alveus.

Beyond neuronal degeneration, astrocytes have also been implicated in the pathogenesis of AD due to their roles in neuroinflammation and oxidative stress (Arranz and De Strooper, 2019; González-Reyes et al., 2017). Under normal physiological conditions, these glial cells support neuronal survival through regulation of calcium signaling, glutamate clearance, extracellular potassium buffering, and energy metabolism. However, these functions become compromised during AD progression, despite astrocytes exhibiting early neuroprotective responses (Acosta et al., 2017).

To gain better insight into the pathogenesis and progression of neurodegenerative diseases, several experimental models have been developed. Among these, scopolamine hydrobromide is widely employed in neuroscience research to induce neurodegeneration by mimicking the cellular and cognitive features of dementia in experimental models (Chen and Yeong, 2020). This antimuscarinic agent crosses the blood brain barrier, aims at the cholinergic system and impairs memory by promoting oxidative stress, causing chronic inflammation, elevating tau protein phosphorylation and increasing the deposition of Aβ protein all leading to cell death. These are hallmarks of Alzheimer’s type of dementia (Kaur et al., 2015; Mousavizadeh et al., 2022). In addition to neuronal damage, scopolamine-induced oxidative stress and inflammatory processes have also been implicated in astrocytic dysfunction. Although the effect of scopolamine on astrocytes is indirect, the inflammation, oxidative stress, and protein misfolding triggered by scopolamine compromise astrocyte function, thereby making these glial cells critical contributors to the pathogenesis of AD (Rodríguez-Giraldo et al., 2022).

This antimuscarinic agent has also been reported to affect myelin sheath but indirectly by causing a significant decrease in the expression of the myelin basic protein (MBP), a key component of the myelin sheath. In a study by Park et al. (2016) and Chen et al. (2018) the chronic administration of scopolamine decreased the density of MBP-immunoreactive myelinated fibers in the hippocampus of mice, 2 weeks after treatment. The reduced density indicated a degradation of the protein (MBP) essential for the forming and maintaining of myelin sheath therefore linking the toxic effect of scopolamine on neuronal degeneration via myelin sheath constituent insufficiency.

The main goal of medications used to manage of neurological conditions is to alleviate symptoms, slow down the development of the disease and improve the quality of life of affected individuals (Monteiro et al., 2017; Passeri et al., 2022). However, the medications used to manage AD and other neurodegenerative disorders have side effects, are expensive, have limited efficacy, and have limited rural access. Consequently, there has been increasing interest in the exploration of medicinal plants as alternative therapeutic agents due to their accessibility, affordability, and long-standing use in traditional medicine (Ayoka et al., 2005).

One medicinal plant that has gained scientific attention for its therapeutic potential is *Spondias mombin* known as Hog plum (Figure 1a). *Spondias mombin* is a flowering tree of the family Anacardiaceae, naturalized in parts of Africa, South America, as well as a number of global tropical forests in the world (Ogunro et al., 2023). This deciduous fruit tree is known by several local names in Nigeria, including Iyeye (Yoruba), Tsadermasar (Hausa), and Ijikara (Igbo). Researches have uncovered that the various parts of this plant have various therapeutic benefits. Plant parts like the stem, leaves, flowers, bark, fruits, seeds, and root are viable in managing diarrhea, dysentery, inflammation, stomach ache, biliousness, hemorrhoids, tuberculosis, memory enhancement, antidiabetic and many other conditions (Ogunro et al., 2023). Based on the phytochemistry of this plant, steroids, flavonoids, alkaloids, glycoside, terpenoids, tannins and saponins are the bioactive compounds responsible for its therapeutic benefit (Eze-Steven et al., 2019; Omoboyowa et al., 2023).

Given the rich phytochemical content of *Spondias mombin*, neuroscience research indicates that *Spondias mombin* exhibits notable neuroprotective properties, largely attributed to its strong antioxidant capacity and its ability to modulate key enzymes associated with neurodegenerative diseases (Ishola et al., 2018; Eze-Steven et al., 2019; Ademosun et al., 2022; Ojo et al., 2021).

Despite growing evidence supporting the neuroprotective properties of *Spondias mombin*, limited information exists regarding its effect on scopolamine-induced neurodegenerative changes involving astrocytes, hippocampal cytoarchitecture, and myelin integrity. Therefore, this study aimed to investigate the neuroprotective effects of the ethanol extract leaves of *Spondias mombin* (ELESM) against scopolamine-induced neurodegeneration in adult male Wistar rats.

## Materials and methods

### Plant collection and authentication

Fresh leaves of *Spondias mombin* were obtained from Modomo quarters, Ile-Ife, Osun state (7°50’ N, 4°50′E). Plant leaves (fresh) were authenticated by a taxonomist in the Department of Botany, Obafemi Awolowo University, Ile-Ife. A voucher specimen number IFE/18476 was assigned, and the plant was deposited in the departmental herbarium for future references.

### Preparation of ethanol extract of *Spondias mombin* leaves

Following the methods of Ishola et al. (2018) *Spondias mombin* leaves were air-dried, weighed and pulverized. Thereafter the pulverized leaves were soaked in 70 percent ethanol for 72 h at room temperature with intermittent agitation using magnetic stirrer. The obtained extract was filtered using Whatman number 1 filter paper, and the filtrate was concentrated in a rotary evaporator and further freeze dried till a deep green extract was obtained. The obtained yield was 6.25% which was calculated as $\frac{\text{weight of obtained extract}(g)}{\text{weight of pulverized}\;\mathrm{SM}\;\text{leaves}\;(g)}\;X\;100.$

### Drugs

- Scopolamine procured from Sigma and Aldrich chemicals Co (St. Louis, MO, USA)
- Donepezil Hydrochloride (RANBAXY 10 mg) procured from Mohaz pharmaceuticals LTD

Before the experiments, scopolamine, donepezil and ELESM were dissolved in normal saline solution before administration.

### Animal care

Forty-two adults male Wistar rats with weight range of 200 ± 20 g were used for this study. The rats were procured from the Animal Holding of College of Health Sciences, OAU, Ile-Ife. The rats were housed in wire-plastic cages, kept under natural laboratory conditions of temperature, humidity, and light; fed on standard laboratory rat chow and given unrestricted access to clean water. Ethical clearance was obtained from the Health Research and Ethics Committee (HREC) of the Institute of Public Health, Obafemi Awolowo University, Ile-Ife (IPH/OAU/12/2890).

### Experimental design

The study was conducted over a period of 71 days, during which the animals were randomly assigned into six groups of seven rats each. Group 1 received normal saline for a duration of 10 weeks, whereas neurodegeneration was induced in groups 2–6 by administering scopolamine intraperitoneally for 2 weeks. 24 h post scopolamine treatment, group 2 was treated with normal saline, Donepezil was administered orally to group 3 and ELESM was administered orally to groups 4, 5 and 6 at doses of 200 mg/kg, 400 mg/kg and 600 mg/kg, respectively. This treatment continued for a period of 8 weeks (Table 1).

**Table 1:** Experimental design and treatment protocol.

Group (*N* = 7)	Dosage and treatment	Route of administration	Duration (days)
I	Normal saline only (1 mL/kg)	Oral (P.O.)	70
II	Scopolamine (1 mg/kg) + Normal saline (1 mL/kg)	Intraperitoneal (I.P.) + P.O.	14 + 56
III	Scopolamine (1 mg/kg) + Donepezil (5 mg/kg)	I.P. + P.O.	14 + 56
IV	Scopolamine (1 mg/kg) + ELESM 200 mg/kg	I.P. + P.O.	14 + 56
V	Scopolamine (1 mg/kg) + ELESM 400 mg/kg	I.P. + P.O.	14 + 56
VI	Scopolamine (1 mg/kg) + ELESM 600 mg/kg	I.P. + P.O.	14 + 56

### Neurobehavioral test

The behavioral tests were performed 24 h after drug/extract administration (day 71). The rats were accustomed to both open field test (OFT) and Y-maze prior to that the procedure. To eliminate scent left by previous subject rat, both apparatuses main chamber was wiped with 70% ethanol and allowed to evaporate to prevent experimental bias. The experiment duration was 5 min and activities were recorded with a camera (Logitech C270 Hd Webcam, 720p).

The open field test evaluates, exploration, and anxiety-like behaviors in animals. The four walled wooden apparatus (square shaped) has one of its sides as a clear Plexiglas that allows rat visibility. The field consist of 16 squares with a centre square which is marked with a different color to differentiate from the 16 squares. The parameters assessed include line crossings, number of rearing activities, immobility time and the number of fecal boli. At the onset of the experiment, all rats were placed in one of the 16 squares which served as the starting point.

The Y-maze test assesses spatial memory function by observing animal navigation through the maze, and measured as percentage spatial alternation behavior (%SAB). The wooden maze consists of 3 arms (ABC), the rats were placed at the start of one arm and the sequence and number of entries was noted. An entry was counted when the hind paws of the rat were completely within the arm while an actual alternation was defined when a rat entered into all three arms of the maze (i.e ABC, BAC, CBA, BCA or CAB but not CAC, ACA or BAB). Spontaneous alternation was calculated as $\frac{\text{Actual alternation made}\;\mathrm{by}\;\mathrm{rat}}{\text{Number of}\;\mathrm{arm}\;\text{entries}-2}\times 100.$ Spontaneous alternation behavior is considered to reflect spatial working memory a form of short-term memory (Ionita et al., 2017; Memudu and Adanike, 2022).

### Determination of body weight change

The body weight of the rats were recorded twice weekly throughout the experimental period using a top loader weighing balance.

### Animal sacrifice

After the neurobehavioral assessment, rats were fasted overnight (to minimize metabolic variability and ensure accurate neurochemical findings) then euthanized by cervical dislocation, thereafter the heads were severed from the neck and a midline scalp incision was made to reflect the skin so as to expose the skull. Fine scissors was used to cut along the sagittal midline suture from the foramen magnum region forward toward the frontal bone and the bones were flapped to expose the brain. Using a spatula, the brain was lifted separating it from the cranial nerves. The excised brain was weighed, halved then fixed in neutral buffered formal saline for histological, histochemical and immunological studies and for biochemical study hippocampus was excised from the other half and tissues were stored in ice for further processing

### Histological procedures

Brain tissues were processed via paraffin embedding method of Drury and Wallington (1980). 5 μm thick tissues were stained with hematoxylin and eosin and Luxol fast blue to assess the general histoarchitecture and the stratum alveus of the hippocampus, respectively.

### Immunohistochemistry

GFAP expression in astrocytes in a formalin-fixed paraffin embedded brain tissue sections were examined using glial IHC protocol adopting the method of Bancroft and Gamble (2006).

### Photomicrograph and image analysis

Tissues were scanned with *Aperio CS2 (ss 5710)*. Image J software (1.53 s; Java 1.8.0_112) was used for histomorphometry analysis such as cell count, neuronal density and relative healthy neurons. For the CA subfield normal/healthy pyramidal neurons were typically identified as cells with round nuclei and clear cytoplasm, whereas degenerating pyramidal neurons appeared flame-like in appearance, darked stained (inability to distinguish between nuclei and cytoplasm) accompanied with pyknosis features. These cells (healthy and unhealthy) were counted manually using a multi-point tool. For the neuronal density in DG subfield a rectangle of area 45,381.250 μm2 (0.045 mm2) was superimposed on the photomicrographs (4 per micrograph) and cells were counted with a multi-point tool. The obtained data was then analyzed using *Graphpad prism* 8.4.3.686. However, preceding the histomorphometry assessment, scale was set using a digital micrometer gauge reading to convert measurements in pixel to microns and this was applied globally to all images.

### Biochemical procedures

The excised hippocampus was homogenized with ice cold 0.1 M phosphate buffer (pH 7.4). The homogenate was centrifuged at 10000 x g for 15 min, the supernatant was separated and aliquots was used for biochemical estimations.

### Estimation of acetylcholinesterase levels

Following the manufacturer’s instructions of the AChE assay kit (E-BC-K174-M), AChE enzymatic activity in the hippocampus was measured and expressed as U/mgprot. The was detected based on the AChE ability to catalyzes the hydrolysis of acetylcholine to form choline, and choline reacts with dithio p-nitrobenzoic acid (DTNB) to form 5-metacarpo-nitrobenzoic acid (TNB). TNB has an absorption peak at 412 nm, and the activity of AChE is calculated by measuring the increasing rate of absorbance of 412 nm.

### Estimation of malondialdehyde levels

The concentration of MDA, a product of lipid peroxidation in hippocampus homogenate was measured using the assay kit (E-BC-K025-M), guided by the manufacturer’s instructions. The concentration was expressed as nmol/gprot., and for its detection, MDA in the catabolite of lipid peroxide react with thiobarbituric acid (TBA) to a produce red compound, its absorbance was read with a spectrophotometer at 532 nm

### Estimation of superoxide dismutase activity

SOD activity was assayed using the SOD activity assay kit (E-BC-K019-S), following the manufacturer’s instructions. The activity of SOD was expressed as U/mg protein. The superoxide anion free radical (O^2−^) produced by xanthine and xanthine oxidase reaction system oxidizes hydroxylamine to form nitrite, a purple reaction. The inhibitory effect of SOD on O^2−^ formation reduce the formation of nitrite hence, the absorbance value of sample tube is lower than control tube (absorption peak at 550 nm).

### Estimation of catalase activity

Catalase activity was measured using a CAT assay kit (E-BC-K031-S), following the manufacturer’s instructions. The activity was expressed as U/mg. To detect activity, the decomposition of H_2_O_2_ by catalase is inhibited by ammonium molybdate, then the residual H_2_O_2_ reacts with ammonium molybdate to generate a yellowish complex. CAT activity can be calculated by production of the yellowish complex at 405 nm. The amount of CAT in 1 mg of tissue protein that decompose 1 μmol H2O2 per minute at 37 °C is defined as 1 unit.

### Estimation of hippocampal TNF-α content

Hippocampal TNF-α concentration was assessed by the sandwich-ELISA principle using ELISA kits (E-EL-R2856, USA) and the results were expressed as picograms of antigen per mL (Shabani and Mirshekar, 2018).

### Statistical analysis

Data obtained were analyzed using one-way analysis of variance (ANOVA) followed by Tukey’s test for multiple comparisons. Results were expressed as Mean ± S.E.M. Alpha level of 0.05 was taken as significant. All computations were performed by GraphPad prism version 8.4.3.686.

## Results

### Body weight assessment

As shown in Figures 2A,B, the body weight trajectory and the percentage change in body weight was assessed across the experimental groups. Statistically no significant differences were observed among these groups. Although the mean values of the experimental groups (2–6) were lower than that of the normal control group (1), this difference was not statistically significant (*p* > 0.05).

**Figure 2:**
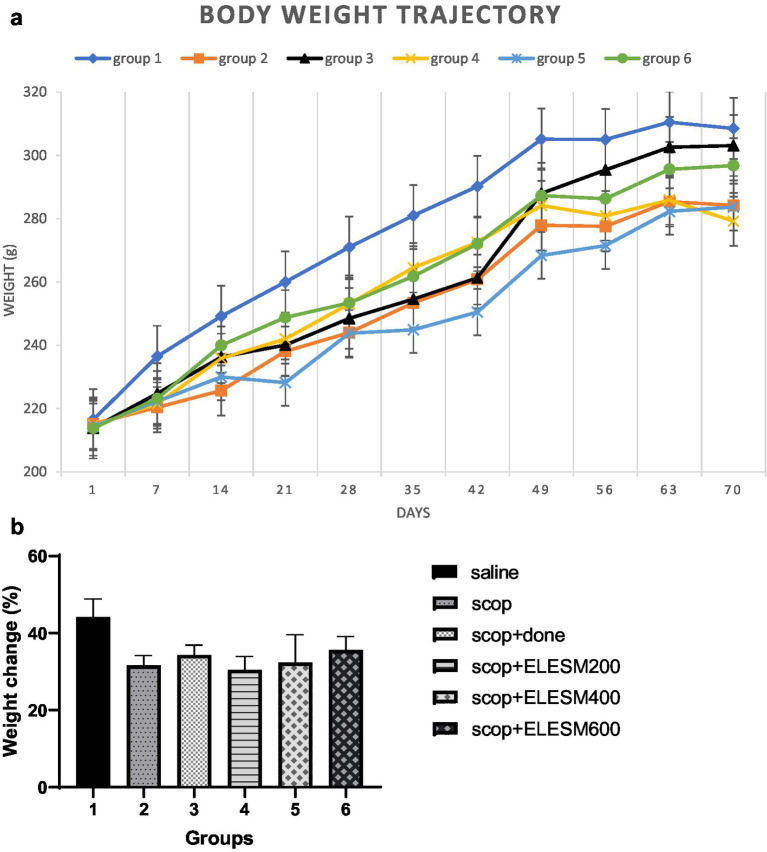
**(a)** Graph showing body weight trajectory. Day 1–14: Scopolamine administration. Day 15–70: ELESM, Donepezil treatment. **(b)** ELESM effect on the percentage change in body weight of scopolamine-induced neurodegeneration in adult male Wistar rats. Each bar represents mean ± SEM, using one way ANOVA, Tukey’s test. Significance value set at *p* < 0.05.

### Cognitive test and memory assessment

Shown in Figure 3 is the open field test graph showing the effect of ELESM on the number of crossings made by adult male Wistar rats with scopolamine-induced neurodegeneration. The number of crossings in group 2 was significantly reduced compared to group 1 (*p* = 0.0153). However, a significant recovery was observed in groups 3, 4, and 5 when compared with group 2 (*p* = 0.0023, *p* = 0.0034 and *p* = 0.0296 respectively). Although group 6 showed an increase relative to group 2, but this change was not statistically significant (*p* = 0.1030).

**Figure 3:**
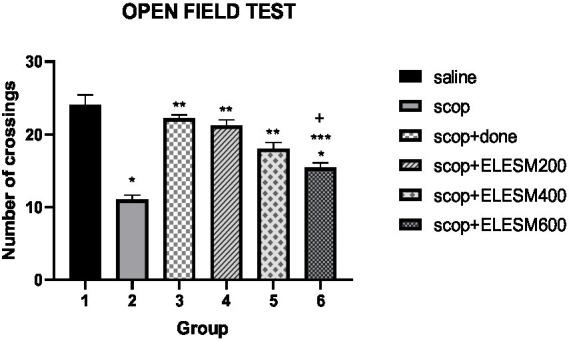
Open field test graph showing the effects of ELESM on the number of crossings made by adult male Wistar rats with scopolamine-induced neurodegeneration. Data are presented as mean ± SEM. ^*^p < 0.05 versus group 1, ^**^p < 0.05 versus group 2, ^***^p < 0.05 versus group 3, and ^**+**^*p* < 0.05 versus group 4, using one way ANOVA, Tukey’s test.

The graph in Figure 4 is an open field test graph showing the effect of ELESM on the immobility time in adult male Wistar rats with scopolamine-induced neurodegeneration. Group 1 spent less time immobile compared to group 2–6. The difference between the normal control and experimental groups (2–6) was found statistically significant (*p* < 0.0001, *p* = 0.0016, *p* = 0.0048, *p* = 0.0015 and *p* < 0.0001 respectively). Immobility time was significantly reduced in group 3 when compared to group 2, 4, 5 and 6 (*p* < 0.0001, *p* = 0.0227, *p* = 0.0045 and *p* = 0.0018 respectively). Furthermore, within the ELESM-treated groups, immobility time was significantly reduced in groups 4 and 5 (*p* = 0.0054 and *p* = 0.0162 respectively) compared to group 2, while group 6 also showed a reduction, although less pronounced (*p* = 0.0507). Notably, an increase in immobility time was observed with increasing doses, indicating a dose-dependent effect during the open field test.

**Figure 4:**
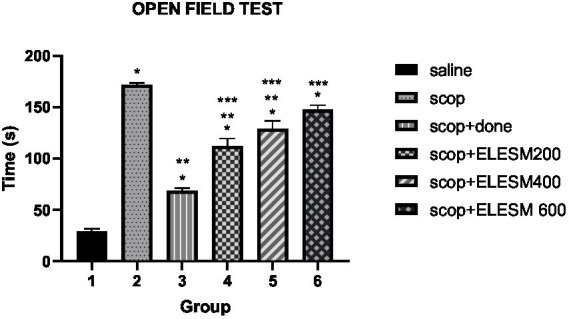
Open field test graph showing the effects of ELESM on immobility time in adult male Wistar rats with scopolamine-induced neurodegeneration. Data are presented as mean ± SEM. ^*^p < 0.05 versus group 1, ^**^p < 0.05 versus group 2, and ^***^p < 0.05 versus group 3, using one way ANOVA, Tukey’s test.

Figure 5 illustrates the effect of ELESM on fecal boli, an indicator of anxiety. Scopolamine administration caused a significant increase in the number of fecal boli in group 2, when compared to group 1 (*p* = 0.0009). In contrast, treatment with donepezil and ELESM 600 mg/kg caused a significant decrease in the number of fecal boli in group 3 (*p* = 0.0477) and 6 (*p* = 0.0264) respectively, when compared to group 2. Although a reduction was also seen in groups 4, 5 but statistically was found not significant relative to group 2 (*p* = 0.1701 and *p* = 0.2436 respectively).

**Figure 5:**
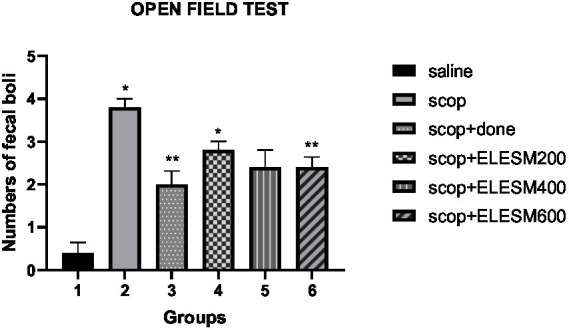
Open field test graph showing the effects of ELESM on fecal boli count in adult male Wistar rats with scopolamine-induced neurodegeneration. Data are presented as mean ± SEM. ^*^p < 0.05 versus group 1, and ^**^p < 0.05 versus group 2, using one way ANOVA, Tukey’s test.

Illustrated in Figure 6 is the effect of ELESM on the number of rearing. Scopolamine administration led to a significant decrease in rearing behavior across all experimental groups (all except group 5) when compared to the normal control group (*p* = 0.0109, *p* = 0.0239, *p* = 0.0239, *p* = 0.0606 and *p* = 0.0050 respectively). However, donepezil caused a significant increase in the number of rearing made by group 3 compared to group 2, indicating recovery. Similarly, ELESM 200 mg/kg, 400 mg/kg, and 600 mg/kg increased the number of rearing in the rats compared to rats treated with scopolamine only, indicating recovery, but not to the level observed in the control group.

**Figure 6:**
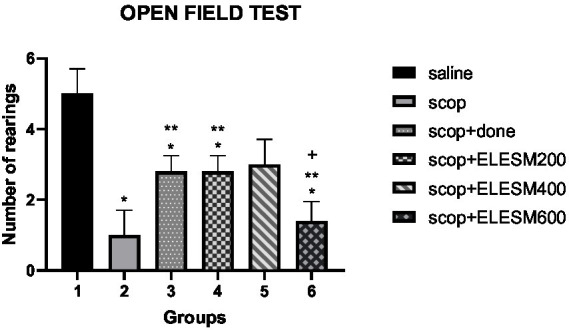
Open field test (rearing) graph showing the effects of ELESM on scopolamine-induced neurodegeneration in adult male Wistar rats. Data are presented as mean ± SEM. *p < 0.05 versus group 1, **p < 0.05 versus group 2, and ^+^*p* < 0.05 versus group 4, using one way ANOVA, Tukey’s test.

Spontaneous alternation behavior (SAB%) was significantly reduced in all groups treated with scopolamine (Groups 2–6) as seen in Figure 7. However, treatment with Donepezil, ELESM 200 mg/kg, ELESM 400 mg/kg and ELESM 600 mg/kg significantly increased SAB% when compared to group 2 treated with scopolamine only (*p* = 0.0018, *p* = 0.0288, *p* = 0.0001 and *p* = 0.0005 respectively) indicating improved memory function. The memory-enhancing effect of ELESM was observed to be dose-dependent (group 4 < group 5 < group 6).

**Figure 7:**
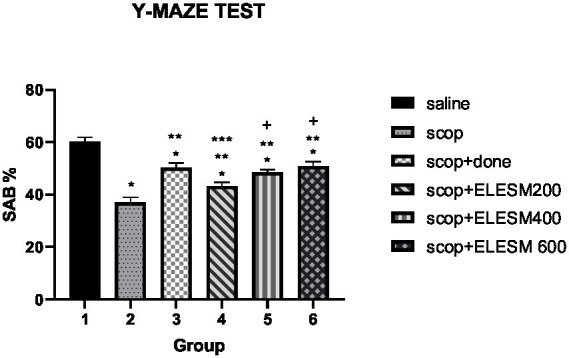
Y-maze test graph showing the effects of ELESM on spontaneous alternation behavior (SAB) in adult male Wistar rats with scopolamine-induced neurodegeneration. Data are presented as mean ± SEM. ^*^p < 0.05 versus group 1, **p < 0.05 versus group 2, ***p < 0.05 versus group 3, and ^+^*p* < 0.05 versus group>p 4, using one way ANOVA, Tukey’s test.

### Biochemical evaluations

#### The effect of ELESM on the acetylcholinesterase activity (AChE)

The graph in Figure 8 illustrates the effect of ELESM on acetylcholinesterase (AChE) activity in scopolamine-induced neurodegeneration. A marked increase in AChE activity was observed in Group 2 when compared to Group 1, and this increase was statistically significant (*p* = 0.0300). However, treatment with Donepezil and ELESM at doses of 200 mg/kg, 400 mg/kg, and 600 mg/kg resulted in a significant reduction in AChE activity when compared to the scopolamine-treated group (*p* = 0.0111, *p* = 0.1716, *p* = 0.0034, *p* = 0.0057 respectively). Although a reduction in AChE activity was observed in Group 4, this decrease was not statistically significant when compared to Group 2 (*p* = 0.1716).

**Figure 8:**
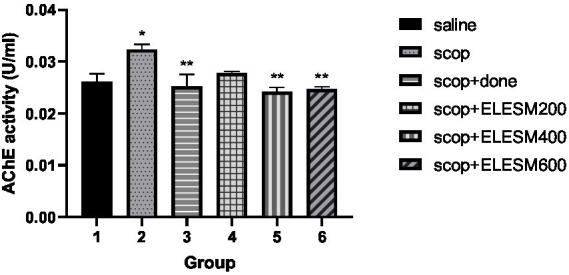
ELESM effect on the acetylcholinesterase activity (AChE) of scopolamine-induced neurodegeneration in adult male Wistar rats. Data are presented as mean ± SEM. *p < 0.05 versus group 1, and **p < 0.05 versus group 2, using one way ANOVA, Tukey’s test.

#### The effect of ELESM on the superoxide dismutase activity (SOD)

Illustrated in Figure 9 is the effect of ELESM on superoxide dismutase (SOD) activity in scopolamine-induced neurodegeneration. A significant reduction in SOD activity was observed in the scopolamine-treated groups (Group 2–6) when compared to the normal control group. Specifically, SOD activity in Group 2 was significantly lower than that of Group 1 (*p* = 0.0116). However, treatment with Donepezil and ELESM resulted in a significant increase in SOD activity across the treatment groups when compared to Group 2, indicating a restoration of antioxidant activity.

**Figure 9:**
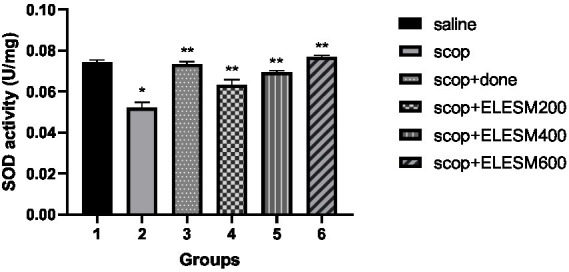
ELESM effect on the superoxide dismutase activity (SOD) of scopolamine-induced neurodegeneration in adult male Wistar rats. Data are presented as mean ± SEM. *p < 0.05 versus group 1, and **p < 0.05 versus group 2, using one way ANOVA, Tukey’s test.

#### The effect of ELESM on catalase activity (CAT)

A pattern similar to SOD activity was observed for catalase activity. Figure 10 illustrates the effect of ELESM on catalase (CAT) activity in scopolamine-induced neurodegeneration. A significant decrease in CAT activity was observed in Group 2 when compared to Group 1 (*p* = 0.0071), indicating impaired antioxidant defense. However, treatment with ELESM and Donepezil significantly increased CAT activity when compared to Group 2, demonstrating an improvement in antioxidant enzyme activity of rats provided with interventions.

**Figure 10:**
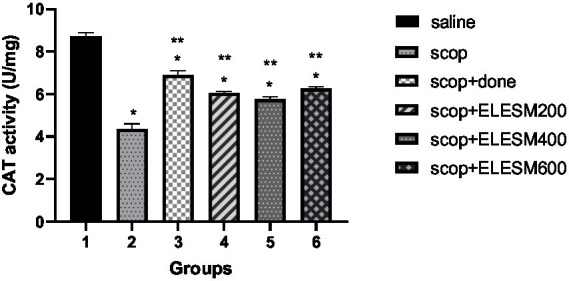
ELESM effect on the catalase activity (CAT) of scopolamine-induced neurodegeneration in adult male Wistar rats. Data are presented as mean ± SEM. *p < 0.05 versus group 1, and **p < 0.05 versus group 2, using one way ANOVA, Tukey’s test.

#### The effect of ELESM on the malondialdehyde concentration (MDA)

Illustrated Figure 11 is the effect of ELESM on malondialdehyde concentration (CAT) of scopolamine-induced neurodegeneration. The MDA concentration of the control group was found significantly lower when compared with both scopolamine only (*p* = 0.0087) and ELESM treated groups. Whereas the low, medium, high doses of ELESM and Donepezil reduced MDA concentration significantly when compared to the scopolamine only treated group (*p* = 0.0248, *p* = 0.0158, *p* = 0.0414 and *p* = 0.0071 respectively). A reduction of MDA concentration was caused by ELESM, however, the efficacy of low dose ELESM treatment was lower compared to donepezil treatment, and the difference was found statistically significant (*p* = 0.0018).

**Figure 11:**
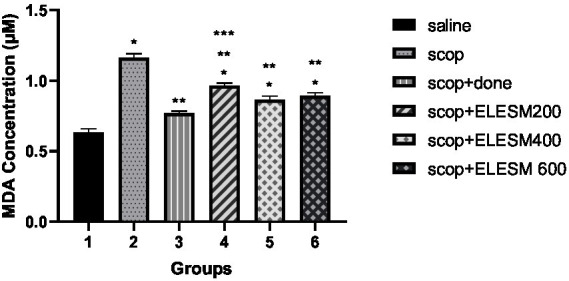
ELESM effect on the malondialdehyde concentration (MDA) of scopolamine-induced neurodegeneration in adult male Wistar rats. Data are presented as mean ± SEM. ^*^p < 0.05 versus group 1, **p < 0.05 versus group 2, and ***p > <0.05 versus group 3, using one way ANOVA, Tukey’s test.

#### The effect of ELESM on the tumour necrosis factor-alpha (TNF-*α*)

Illustrated in Figure 12 is the effect of ELESM on tumour necrosis factor-alpha concentration (TNF-α) of scopolamine-induced neurodegeneration. TNF-α levels in group 2 was significantly elevated when compared to Group 1 (*p* = 0.0046), indicating enhanced inflammatory response, and this elevation persisted throughout the experimental period. However, treatment with ELESM and donepezil reduced TNF-α levels in Groups 3–6 significantly when compared to Group 2 (*p* = 0.0004, *p* = 0.0274, *p* = 0.0022 and *p* = 0.0020). Furthermore, the reduction in TNF-α concentration observed at the highest dose of ELESM (600 mg/kg) was significantly greater than that observed at the lower dose (400 mg/kg) (*p* = 0.0242), indicating a dose-dependent anti-inflammatory effect.

**Figure 12:**
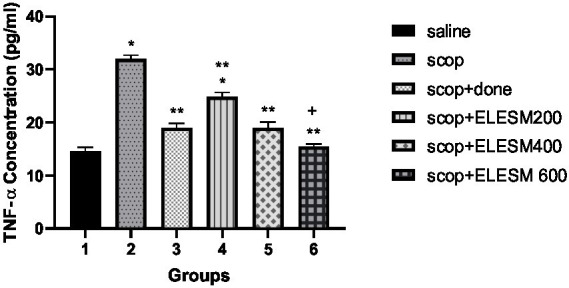
ELESM effect on the tumour necrosis factor-alpha concentration (TNF-α) of scopolamine-induced neurodegeneration in adult male Wistar rats. Data are presented as mean ± SEM. *pp< 0.05 versus group 1, **p < 0.05 versus group 2, and ^+^*p* < 0.05 versus group 4, using one way ANOVA, Tukey’s test.

### Histopathological assessment

Shown in Figure 13 is the histological examination of *H and E-stained* section of the hippocampus (DG) of scopolamine-induced Wistar rat. The normal control group showed intact, well populated granule cell bodies with no obvious sign of cell degeneration. Group 2, Scopolamine only group showed signs of neurodegeneration characterized by reduced neuronal cell population, with cells appearing pyknotic and some identified as necrotic. The interventions (Donepezil and ELESM) preserved tissue integrity and increased neuronal population as seen in groups 3, 4, 5, and 6.

**Figure 13:**
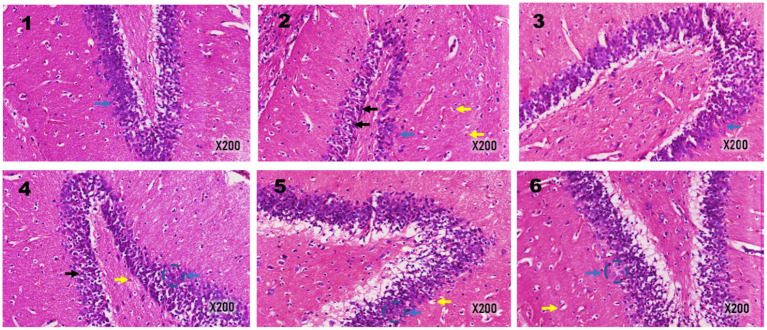
Representative photomicrograph of hippocampus (DG) across groups. The blue arrow shows a relatively normal granule cell body, the black arrow shows pyknotic neuron and the yellow arrow identifies eosinophilic necrosis. H & E X200.

Figure 14 shows the histological examination of *H and E-stained* section of the hippocampus (CA3) of scopolamine-induced Wistar rat. The normal control group shows numerous neuronal cells with prominent nuclei; in contrast the toxic group shows signs of degeneration characterized by reduced cell number and pyramidal cells appearing darkened and flame like in appearance. ELESM treatments appeared to be dose-dependent as seen in groups 4, 5, and 6. Donepezil treatment reversed the damage, restoring cell health to level of normal evident by the numerous nuclei with prominent nucleoli.

**Figure 14:**
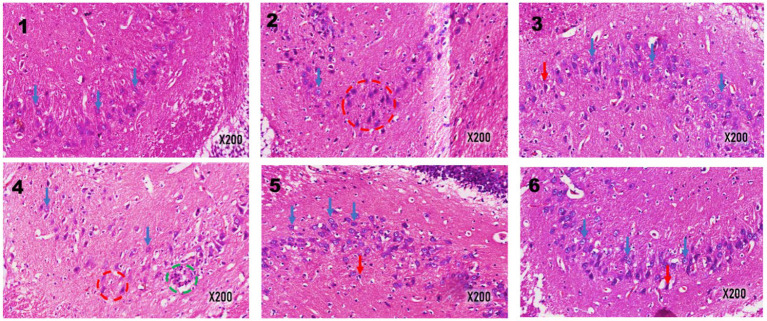
Representative photomicrograph of the hippocampus (CA3) across groups. The blue arrow shows a relatively normal pyramidal cell body, the red arrow identifies darkly stained degenerating cells, the red circle and green identifies a cluster of darkly stained neurodegenerating neurons and focal gliosis, respectively. H & E X200.

Figure 15 shows the histological examination of *H and E-stained* section of the hippocampus (CA1) of scopolamine-induced Wistar rat. The normal control group showed a normal neuronal architecture characterized by a proper cellular organization, and a clearly defined pyramidal neurons with round to oval nuclei. In contrast to the normal control, the scopolamine treated group exhibited some neurodegenerative changes characterized by the presence of dark stained, shrunken and pyknotic neurons in addition with cellular disorganization. Group 3, 4, 5 & 6 showed restored cellular disorganization with the presence of more healthy neurons indicating preservation of neuronal architecture integrity.

**Figure 15:**
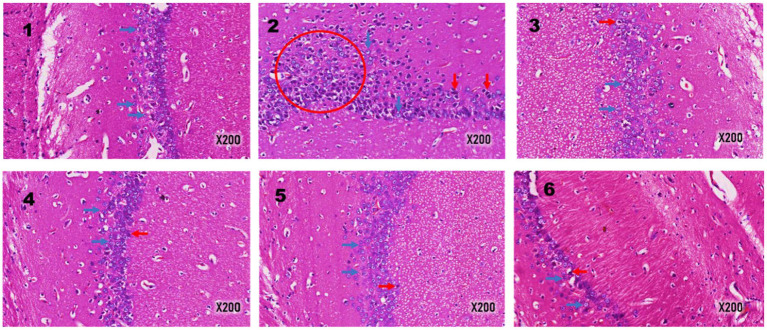
Representative photomicrograph of the hippocampus (CA1) across groups. The blue arrow shows a relatively normal neuron, the red arrow identifies darkly stained degenerating cells, and the red circle shows cellular disorganization with a cluster of degenerating (majority) and healthy neuron. H & E X200.

#### Quantitative morphometric analysis of hippocampal subregions

Quantitative assessment of the hippocampus (Figure 16) revealed that relative to the control group, the population density of granule cells in the DG of scopolamine treated rats was significantly lower (*p* = 0.0042) indicating cellular degeneration, however the administration of intervening agent (Donepezil, ELESM) helped with cell preservation indicating neuroprotection. Similarly, in the CA1 and CA3 subfield (Figures 17, 18 respectively), the percentage of neurons with distinct cell body and nucleus (indicative of healthy neurons) in the scopolamine treated groups was significantly reduced compared to the control group (*p* = 0.0079 and *p* = 0.0382 respectively). However, treatment with ELESM and Donepezil offered cell survival as reflected by the higher percentage of healthy neurons compared to the scopolamine group (*p* < 0.05).

**Figure 16:**
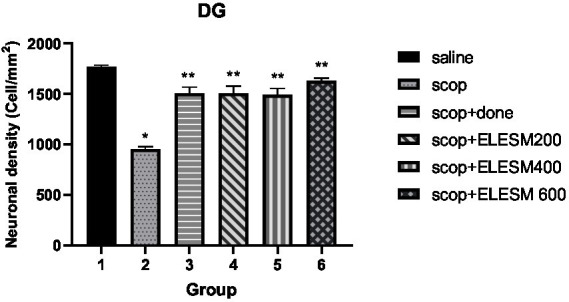
The effects of ELESM on the DG3 region of scopolamine-induced neurodegeneration in adult male Wistar rats. Data are presented as mean ± SEM. *p < 0.05 versus group 1, and **p < 0.05 versus group 2, using one way ANOVA, Tukey’s test.

**Figure 17:**
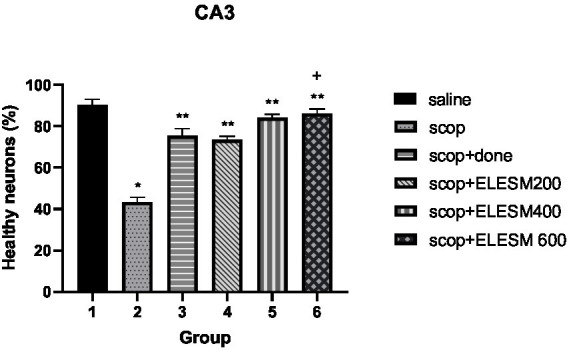
The effects of ELESM on the CA3 region of scopolamine-induced neurodegeneration in adult male Wistar rats. Data are presented as mean ± SEM. *p < 0.05 versus group 1, **p < 0.05 versus group 2, and ^+^*p* < 0.05 versus group 4, using one way ANOVA, Tukey’s test.

**Figure 18:**
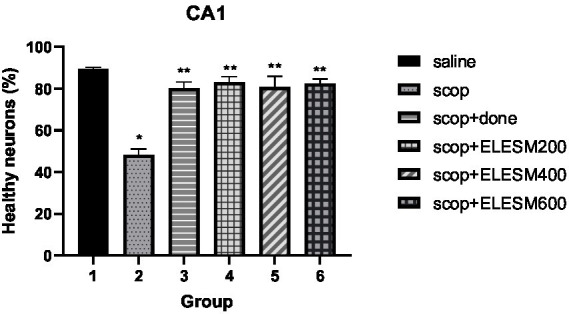
The effects of ELESM on the CA1 region of scopolamine-induced neurodegeneration in adult male Wistar rats. Data are presented as mean ± SEM. *p < 0.05 versus group 1, and **p < 0.05 versus group 2, using one way ANOVA, Tukey’s test.

Figure 19 showed the histological examination of the white matter tract in the alveus using Luxol fast blue. The micrographs reveal a normal tissue integrity in the control group with fibers appearing organized and compacted however, the fibers of the group treated with scopolamine only appear less compacted with signs of fiber disruption and vacuolation suggesting demyelination. Donepezil and ELESM improved alveus fiber integrity promoting compaction as seen in groups 3, 4, 5 and 6.

**Figure 19:**
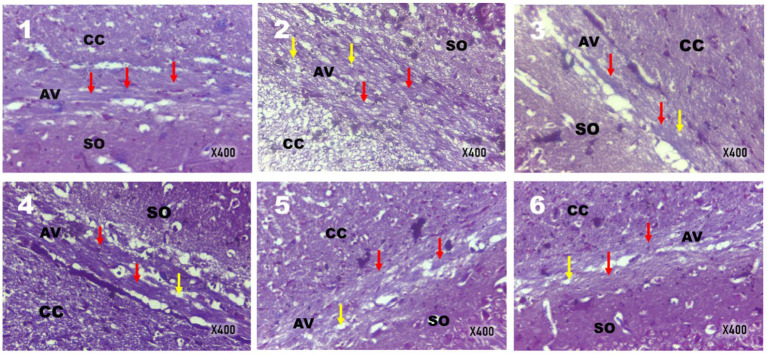
Representative photomicrographs showing the effect of ELESM on the hippocampus. The red arrow identifies white matter tract and the yellow arrow identifies vacuoles. AV-alveus, SO-stratum oriens and CC-corpus callosum. Luxol fast blue X400.

#### Subfield assessment on the effect of ELESM on astrocyte morphology and GFAP expression in the hippocampus of scopolamine treated rats

Figures 20–22 displays micrographs of the hippocampal subfields CA1, CA3 and DG. Micrograph of group 1 in the CA1, CA3 and DG showed less positive GFAP immunoreactivity and astrocytes appear to be in a resting state identified by the small cell body with minimal branching and less thickened processes indicating the absence of insult. In contrast the micrograph of group 2 in the CA1, CA3 and DG displayed intense GFAP immunopositivity characterized by cell hypertrophy and thickened processes and cell clustering indicating astrogliosis in response to insult. ELESM modulated astrocytic reactivity in a dose-dependent fashion with 400 mg/kg and 600 mg/kg producing a result almost similar to the control. In group 4, GFAP immunopositivity was lower compared to group 2, astrocytes appeared less hypertrophied compared to group 2, but the higher dose demonstrated the best outcome indicating a better neuroprotective effect.

**Figure 20:**
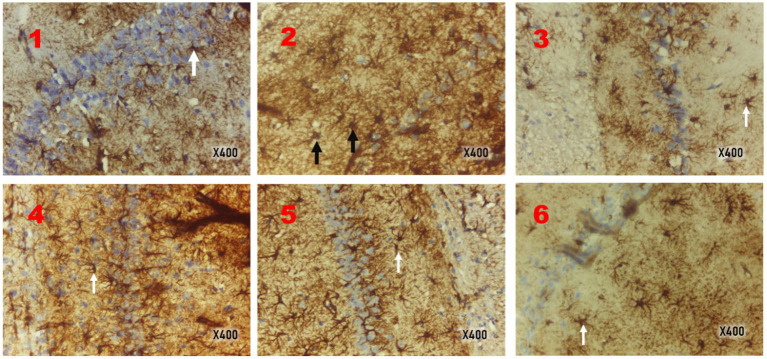
Representative photomicrograph of the hippocampus subfield CA1, showing the effect of ELESM on glial response as detected by GFAP antibody (X400). White arrow - cell atrophy (resting) and black arrow—cell hypertrophy (reactive).

**Figure 21:**
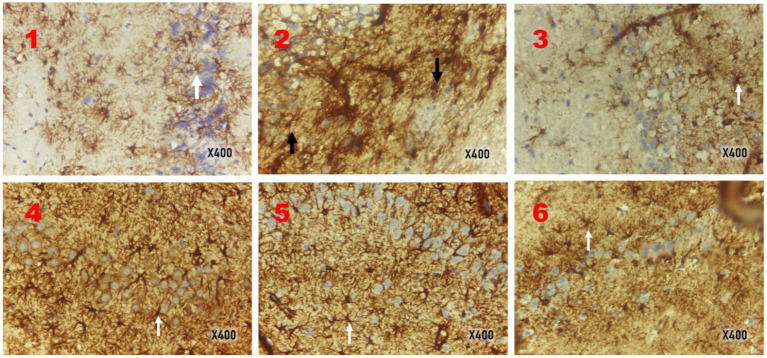
Representative photomicrograph of the subfield CA3 showing the effect of ELESM on glial response as detected by GFAP antibody (X400). White arrow - cell atrophy (resting) and black arrow—cell hypertrophy (reactive).

**Figure 22:**
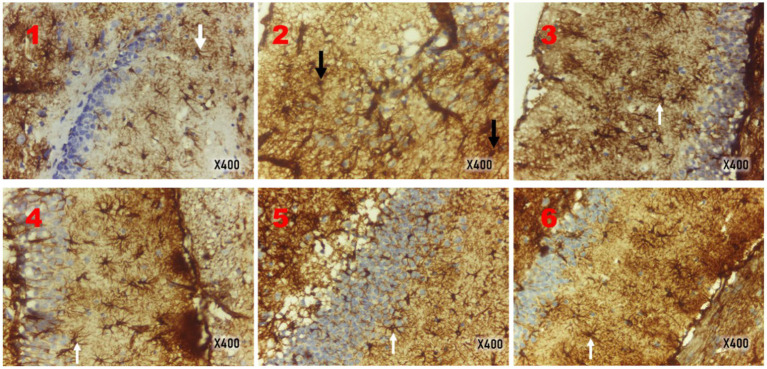
Representative photomicrograph of the hippocampus subfield DG, showing the effect of ELESM on glial response as detected by GFAP antibody (X400). White arrow - cell atrophy (resting) and black arrow—cell hypertrophy (reactive).

## Discussion

The absence of significant differences in body weight across all groups suggests that randomization was effective, as animals in each group began the experiment from a comparable baseline reducing the likelihood of weight gain result influenced by pre-existing weight disparities and ensuring uniformity. However, during scopolamine administration between days 1 to 14, the rate of weight gain was slightly lowered in the experimental groups relative to the normal control group (statistically not significant), meanwhile there was no notable reduction in feed intake and rat chow provided was weighed and constant across all groups. This suggests that scopolamine administration and the stress of handling must have disrupted the normal metabolism and absorption rate in the animals affecting the rate of body weight increase. Research made by the National Toxicology Program (1997) reported that 14-days administration of scopolamine resulted in a significantly lower body weight gain compared to the normal group, however unlike this study, the dose is higher and route is different. There remains a scarcity of study showing that a low dose of scopolamine can reduce weight gain in rodents.

In many studies that focus on the short-term effect, scopolamine is known to alter some biochemical parameters; increasing the level of ROS, diminishing the concentration and activity of antioxidant enzymes resulting in oxidative stress which promote the peroxidation of cell membrane lipids (Kaur et al., 2015; Memudu and Adanike, 2022). These findings are consistent with the findings of this present study. This study revealed a sustained increase in AChE activity, a decrease in antioxidant activity (SOD and CAT), and an elevated level of MDA and TNF-*α* concentration were noted in the hippocampus of group 2 rats, indicating that without intervention and the provided recovery window, scopolamine kick-started a cycle of neurodegeneration that was sustained till the end of the experiment. This is consistent with the findings of Istifo et al. (2024), who proved that the molecular disturbance caused by scopolamine in mice brain tissue is a long-term effect.

The interventions provided help mitigate the toxic effects of scopolamine. Donepezil and ELESM demonstrated both antioxidant and anti-inflammatory properties as seen in the results. This suggests that aside its primary cholinesterase inhibitory effect, donepezil enhanced the antioxidant defense and suppressed the inflammatory signaling. The mechanism of by which donepezil exerts its anti-inflammatory effect is unclear but can be linked to its ability to stop nuclear translocation of NF-kb, suppressing NF-kb signaling pathways (Arikawa et al., 2016). The therapeutic effect ELESM demonstrated in this study, showed that the active phytochemicals in *Spondias mombin* are capable of scavenging the free radicals, enhancing endogenous defense enzymes, and suppressing pro-inflammatory cytokines by interrupting the inflammatory pathway, restoring health to almost normal with ELESM 600 mg/kg showing the most benefit with no physical side effect observed in rats across all groups.

The findings of previous studies align with the result of this study. Nworu et al. (2011) reported that the methanolic leaf extract of *Spondias mombin* has an anti-inflammatory property based on the ability to reduce carrageenan-induced paw edema in a dose-dependent manner with a decrease of TNF-α levels and the inhibition of nitric oxide formation. Similarly, Adetuyi et al. (2024) reported that *Spondias mombin* leaf extract significantly reduced neuroinflammation by downregulating elevated TNF-α expression in the hippocampus and cerebral cortex of aluminium chloride treated rats. In addition, hydroethanolic leaf extract of *Spondias mombin* (HESM) at 200 mg/kg was potent enough to counter oxidative stress and reduce inflammatory markers induced by 5-fluorouracil-induced oral mucositis in hamsters. These therapeutic effects are linked to the phytochemicals present in ELESM (Sousa Gomes et al., 2020).

The sustained AChE hyperactivity in group without intervention is likely due to an implicative effect of reactive astrocytes and microglial in response to stressors. The findings of Melo et al. (2002) established the relationship between oxidative stress, inflammation and the AChE activity. In response to oxidative stress induced by amyloid plaques, AChE activity is increased. This hyperactivity affects the cholinergic anti-inflammatory pathway, a pathway that serves as a regulator (inhibitory effect) of NF-kB activation and pro-inflammatory gene expression in glial cells by preventing NF-kb nuclear translocation.

Study by Rosas-Ballina and Tracey (2009) has shown that acetylcholine is an important neurotransmitter that help with modulating inflammation via binding to the α7 nicotinic receptor which is the reason why cholinesterase inhibitors are administered to reduce inflammation via Inhibitor of kappa B protein upregulation and Nuclear Factor kappa-light-chain-enhancer of activated B cells (NF-kb) protein retention in the cytoplasm. This justifies the therapeutic effect of donepezil demonstrated in *Figure* *3* showing that donepezil helped reduce AChE activity which indirectly reduced inflammation and oxidative stress. Regarding ELESM, its effects on AChE activity demonstrated it anticholinesterase effect, therefore indicating that ELESM most likely suppressed inflammation in a similar pathway with donepezil, restoring the cholinergic inflammatory pathway. This inhibitory effect demonstrated by ELESM is consistent with the findings of Akinmoladun et al. (2021), who reported that *Spondias mombin* facilitated the inhibition of AChE activity and removed the restriction imposed on the cholinergic system.

The rich constituent of this plant has prompted the need to assess it ameliorative potential on a cognitive and memory impairment caused by scopolamine via behavioral studies. Scopolamine causes a cholinergic dysfunction primarily which indirectly triggers several molecular mechanisms that precede neuronal cell death. Behavioral study outcomes of rats treated with scopolamine include hypermotility, anxiety, reduced animal exploration and amnesia. However, many existing studies assess for the short term of scopolamine on cognitive function, in contrast, this study allows a long-term recovery window to determine whether scopolamine induced neurotoxicity is a transient or a long-lasting effect.

The reduction in locomotor and exploratory activities observed several weeks after scopolamine administration suggests that its neurotoxic effects extend beyond acute exposure, leading to sustained impairments in cognitive and behavioral functions. The decrease in spontaneous alternation in the Y-maze further supports the notion of prolonged disruption of working memory, indicating that scopolamine-induced cholinergic dysfunction may have long-lasting consequences in the absence of therapeutic intervention.

The observed improvement following donepezil treatment is in line with its proven effects as an acetylcholinesterase inhibitor, which increases cholinergic neurotransmission and thus improves cognitive impairment. Improvement of the exploratory behavior and performance of working memory confirms the significance of cholinergic pathways in learning and memory functions as it is commonly reported in the neurodegenerative models. ELESM also demonstrated its neuroprotective potential by improving cognitive function, however its low efficacy compared to donepezil suggests a weaker cholinesterase inhibitory effect or that its activity is predominantly mediated through antioxidant and anti-inflammatory mechanisms.

ELESM caused a decrease in the activities of these rats suggesting that it could reflect a mild CNS depressant or sedative effect (side effect). These Findings can be linked to the findings of Ayoka et al. (2005). The authors demonstrated that the methanol, ethanolic and aqueous extract of *Spondias mombin* (≤100 mg/kg) prolonged sleeping time in rats induced with hexobarbital, concluding that *Spondias mombin* is a GABA(A) agonist hence a justification for its sedative effect (Ayoka et al., 2005). This side effect was also reported by Asuquo et al. (2013). It was reported that ELESM reduced alertness in rats, resulting in decreased locomotor activity in an open field test. This effect was attributed to the presence of phytochemicals such as sterols, flavonoids, saponins and tannins identified in *Spondias mombin* leaf extract, which have also been reported in other plant extracts with sedative effects.

Importantly, the observed sedative effect does not necessarily contradict the cognitive and neurobehavioral improvements recorded in this study. The reduction in locomotor or exploratory activity likely reflects mild CNS depressant activity rather than impaired learning or memory. Several neuroprotective agents (including *Spondias mombin*) with anxiolytic or sedative properties have also been shown to improve neurobehavioral outcomes by reducing oxidative stress, neuroinflammation, and neuronal degeneration (Santos et al., 2019; Ogunro et al., 2022; Olasehinde and Olaokun, 2024). Therefore, although ELESM reduced spontaneous activity levels, the extract concurrently preserved neuronal integrity and improved cognitive performance, suggesting that its mild sedative effects did not interfere with its neuroprotective actions.

The restoration of the antioxidant defense system, suppression of inflammation and the reestablishment of the cholinergic function as seen in the biochemical study correspond with the cognitive improvement and enhanced memory function seen in the rats treated with interventions compared to the toxic group. These findings imply that the interventions promoted hippocampal recovery of these animals evident by the biochemical assays and the behavioral assessments.

The histopathological assessment of the hippocampal subfields (CA1, CA3 and DG) and its morphology provides insights into extent of damage caused by oxidative stress and Inflammation, based on tissue integrity and the degree of protection provided by therapeutic interventions across all experimental groups. Histological alterations observed across the hippocampal subfields (CA1, CA3, and dentate gyrus) highlight the vulnerability of these regions to scopolamine-induced neurotoxicity. The hippocampus plays a central role in learning and memory, and the structural disruptions noted in this study suggest a widespread impairment of hippocampal integrity. The degenerative features observed in the hippocampus subfields may be attributed to cholinergic dysfunction, oxidative stress, and inflammatory processes.

The susceptibility of the CA1 and CA3 regions, both critical for memory processing and pattern completion, may be linked to their high metabolic demand and sensitivity to excitotoxic and oxidative insults. Similarly, the DG, which is essential for neurogenesis and memory encoding, appears particularly affected, suggesting that scopolamine exposure may impair neuronal turnover and plasticity. Such degeneration has been strongly associated with diseases such as AD.

Reported in the works of Seifhosseini et al. (2011) and Nikmahzar et al. (2018), scopolamine reduced hippocampal pyramidal and granule cells population, and these findings are consistent with the findings of this study, which explains the behavioral deficit observed in this study. In addition, from the findings of Yan et al. (2014) chronic administration of scopolamine interferes with neurogenesis of nerve cells in adult mouse, this is likely a contributing factor to the reduced cell density seen in the toxic group.

The reduced cell density and number of healthy neurons in the hippocampal subfield are consistent with the findings of Agboola et al. (2024). This indicates that scopolamine initiated a cycle of neurodegeneration that continued into the recovery window (long-term effect), and self-recovery was impossible, indicating the need of an intervention to cease, but through the anti-inflammatory, antioxidant, and cholinesterase inhibitory role of the interventions, cell survival was ensured, which justifies the improvement of cognitive behavioral function seen in the treated rats.

Myelinated axons of pyramidal cells from within the CA subfields with some contributions from the subicular complex forms a thin tract of white matter called the alveus, which tapers to form the fimibrae-fornix pathway. The alveus connects the hippocampus to the limbic and cortical regions (feeds into the papez circuit), serving as a conduit for memory transfer, processing and consolidation. A significant degeneration of CA pyramidal cells and a loss of its axonal projections into the alveus can therefore impair the process of memory encoding and retrieval (Fogwe et al., 2023).

Aside from the cell death induced by scopolamine via biomolecular disturbances and neurotransmission dysfunction, scopolamine is known to disrupt myelin sheath integrity. Chronic treatment of scopolamine has been reported to reduce significantly the expression and density of myelin basic protein in the hippocampus, a protein that comprises of approximately 30% of the total protein content of CNS myelin (Achiron and Miron, 2007; Park et al., 2016; Chen et al., 2018). MBP is essential for neurodevelopment and its primary function is to facilitate myelin sheath compaction. MBP is a positively charged protein that bind to negatively charged lipids such as phosphatidylinositol-4,5-biphosphate [P1(4,5) P2] and its interaction with lipids and other myelin proteins, help organize and compact myelin membranes (Schmitt et al., 2014). It was reported that in shiverer mouse model, loss or mutation of MBP expression resulted in abolished myelin compaction and severe CNS demyelination, however the PNS is not affected (Achiron and Miron, 2007; Xia et al., 2020).

The alveus of the scopolamine treated group exhibited a disruption in myelination appearing less compacted compared to the control, with some identified vacuolated areas indicating a structural compromise. These changes denote demyelination and edema, which is likely linked to the induced oxidative stress caused by scopolamine. This result affirms the findings of Park et al. (2016) and Chen et al. (2018) on the effect of scopolamine on MBP expression and density. The loss of MBP (a protein responsible for compaction) reported by the authors explains the loss of compaction seen in the toxic group and the inability to restore fiber structure integrity can be linked to the ongoing biochemical disturbances (SOD^↓^, CAT^↓^, AChE^↑^, MDA^↑^& TNF-α^↑^) induced by scopolamine. According Kemp et al. (2016) there is an inverse correlation between MBP level and lipid peroxidation products concentration. That is the upregulation of MDA or 4-HNE levels leads to a downregulation of MBP level, and this relates the biochemical and the histological (LFB stain) findings of this study. The loss of CA neurons whose axons contribute to alveus formation, is suggested to be a contributing factor for the reduced compaction observed in this region, thereby impairing memory function.

However, the amelioration of oxidative stress, inflammation, and cholinergic dysfunction by donepezil and ELESM appears to have contributed to the restoration of alveus structural integrity, with higher doses demonstrating greater preservation and reduced evidence of demyelination. This suggests that the bioactive compounds in ELESM must have improved MBP expression/density, thereby contributing to the restoration and preservation of hippocampal myelinated fibers and function.

Oxidative stress and cholinergic stress induced by scopolamine was further confirmed by the activation of astrocytes in rats’ hippocampus. Astrocytes are star shaped cells that happen to be the most abundant of glial cells in the CNS, with the function of neuronal homeostasis, regulation of blood–brain barrier and the facilitation of synapse formation (Wei and Morrison, 2023). In the hippocampus are specific type of astrocytes called protoplasmic astrocytes which are large cells with a high density of tortuous, bulbous GFAP-positive processes that branch into smaller, finer processes and they are found in grey matters (Oberheim et al., 2012). Under conditions of oxidative stress and cholinergic stress, these astrocytes become reactive and undergo distinct cellular changes, including cellular hypertrophy, thickening of branches and upregulation of a major intermediate filament protein, GFAP with the goal of containing neuronal injury and repair (Wei and Morrison, 2023). However, under chronic conditions, prolonged astrogliosis contribute to neuroinflammation and glial scarring (Pekny et al., 2016). Therefore, to assess the potential neuroprotective effect of ELESM, astrocyte activation via GFAP immunoreactivity was assessed to provide insight on ELESM regulation of biomolecular stress.

From the findings of this study, it was observed that the scopolamine treated group displayed intense GFAP immunopositivity characterized by cell hypertrophy with thickened processes, consistent with gliosis. This observation confirms earlier reports that in response to the cholinergic dysfunction, scopolamine initiates oxidative stress and inflammation, and this confirms the earlier statement that a cycle of neurodegeneration was initiated by scopolamine which persisted in the absence of intervention, demonstrating a long-term effect. Conversely, hippocampus of rats treated with ELESM revealed a marked attenuation in GFAP immunoreactivity in a dose-dependent manner with the higher doses yielding the best result. The modulation of astrocyte reactivity by ELESM demonstrate ELESM neuroprotective effect via biochemical imbalance modulation which was confirmed by the biochemical findings of this study (SOD^↑^, CAT^↑^, AChE^↓^, MDA^↓^ & TNF-α^↓^). The effect was comparable to that observed in the donepezil treated group, thereby supporting the potential of ELESM to mitigate astrocyte activation similarly to standard AChE inhibitors.

Regionally, the low GFAP intensity within the CA subfields explains the preservation of pyramidal neurons that are typically vulnerable to oxidative stress and other excitotoxic insults. Furthermore, the DG, an area neurogenesis compromise, showed a restoration of astrocyte cell morphology indicating a possible reversal of the impaired neurogenic processes, justifying the preservation of granule cell bodies seen in H and E. The findings of this study align with previous evidence that plant extracts rich in polyphenols and flavonoids can modulate neuroinflammation by down regulating GFAP expression and attenuating oxidative damage (Ademosun et al., 2022; Chen et al., 2022).

## Conclusion

This study concluded that ELESM exerts neuroprotective effects against scopolamine-induced neurodegeneration, likely mediated through its anticholinesterase, antioxidant, and anti-inflammatory properties in adult male Wistar rats.

## Data Availability

The raw data supporting the conclusions of this article will be made available by the authors, without undue reservation.
